# Prenatal Exposure to Lamotrigine: Effects on Postnatal Development and Behaviour in Rat Offspring

**DOI:** 10.1155/2014/163459

**Published:** 2014-04-14

**Authors:** Sekar Sathiya, Murugan Ganesh, Periyathambi Kalaivani, Vijayan Ranju, Srinivasan Janani, Bakthavachalam Pramila, Chidambaram Saravana Babu

**Affiliations:** ^1^Centre for Toxicology and Developmental Research (CEFT), Sri Ramachandra University, Chennai, Tamil Nadu 600116, India; ^2^Department of Biochemistry, Sri Ramachandra University, Chennai, Tamil Nadu 600116, India; ^3^School of Chemical and Biotechnology, Shanmugha Arts, Science, Technology and Research Academy (SASTRA University), Thanjavur, Tamil Nadu 613402, India

## Abstract

Use of antiepileptic drugs (AEDs) in pregnancy warrants various side effects and also deleterious effects on fetal development. The present study was carried out to assess the effects of prenatal exposure to lamotrigine (LTG) on postnatal development and behavioural alterations of offspring. Adult male and female Sprague Dawley rats weighing 150–180 g b. wt. were allowed to copulate and pregnancy was confirmed by vaginal cytology. Pregnant rats were treated with LTG (11.5, 23, and 46 mg/kg, p.o) from gestational day 3 (GND 3) and this treatment continued till postnatal day 11 (PND 11). Offspring were separated from their dam on day 21 following parturition. LTG, at 46 mg/kg, p.o, produced severe clinical signs of toxicity leading to death of dam between GND 15 and 17. LTG, at 11.5 and 23 mg/kg, p.o, showed significant alterations in offspring's incisors eruption and vaginal opening when compared to age matched controls. LTG (23 mg/kg, p.o) exposed female offspring expressed hyperactive behaviour and decreased GABA-A receptor expression when compared to control rats. These results reveal that prenatal exposure to LTG may impart differential postnatal behavioural alterations between male and female rats which paves way for further investigations.

## 1. Introduction

Use of antiepileptic drugs (AEDs) during pregnancy presents the dilemma of minimising the risk of seizure and avoiding adverse effects in the unborn child. Various studies revealed that prenatal exposure of AEDs imparts serious effects on infants [1–3]. Further, prenatal exposures to AEDs were also reported to produce cognitive impairment [4, 5]. Vigabatrin and valproate were reported to produce neuronal migration defect [6] upon prenatal exposure. Janz and Fuchs [7] reviewed that exposure to AED during pregnancy increases the risk of miscarriage and stillbirth rate. However, little attention was paid to the cellular effects of AEDs during postnatal development and in adulthood.

Lamotrigine (LTG), a phenyltriazine derivative, is one of the most widely used second-generation antiepileptic agents used for both partial and generalised seizures. In pregnant women, LTG is reported to produce side effects such as rash, mania, memory and cognitive problems, mood changes, runny nose, cough, nausea, indigestion, abdominal pain, weight loss, vaginitis, and leukopenia and retardation in development of fetus [8–10]. Recently, exposure to LTG during pregnancy was shown to impart adverse outcome within different developmental domains [11]. On the other hand its use in pregnancy is associated with risk of seizure deterioration, because its clearance is accelerated in pregnancy [12]. This reveals the need of additional information on the therapeutic window of LTG or its possible toxic effects in pregnancy.

Prenatal exposure to LTG (5–20 mg/kg/day) alone and in combination with MX-801, phenobarbital, or phenytoin resulted in cell death in the neonatal rat brain [13]. But, Katz et al. [14] showed no such effects in the same corresponding dose. Thus the effects of LTG during pregnancy, offspring development, and behaviour are still needed to be studied. The present study was undertaken to investigate the effects of prenatal exposure of LTG on postnatal development and its impact on offspring behaviour in rats.

## 2. Materials and Methods

### 2.1. Chemicals and Reagents

LTG was a kind gift from M/s Sun Pharmaceutical Pvt. Ltd., Mumbai, India. Carboxymethyl cellulose (CMC) was procured from Himedia, Mumbai. PCR master cycler gradient was purchased from Genet Bio, Korea. TRIzol Reagent was purchased from Sigma Aldrich, USA. Unless mentioned, all other chemicals and reagents were of analytical grade.

### 2.2. Animal Husbandry and Ethics Approval

20 male and 36 female Sprague Dawley rats were used for the study. Animals were housed in polypropylene cages in a well-ventilated room (air cycles: 12–15 air changes/min, recycle ratio: 70 : 30) under an ambient temperature of 23 ± 2°C and 40–65% relative humidity, with an artificial photoperiod of 12 h light/dark cycle. They were provided with rodent feed (Provimi Animal Nutrition India Pvt. Ltd.) and purified water* ad libitum*. Animals were acclimatized for a period of 7 days to the laboratory conditions prior to initiation of the experiment. Guidelines of “Guide for the Care and Use of Laboratory Animals” (Institute of Laboratory Animal Resources, National Academic Press 1996, NIH publication number 85-23, revised 1996) were strictly followed throughout the study. Institutional Animal Ethics Committee (IAEC), Sri Ramachandra University, Chennai, India, approved the study protocol (IAEC/XIV/SRU/99/2008).

### 2.3. Groups and Treatment

Following acclimatization, female rats were introduced with proven breeder during night time (18:00) and were isolated during day hours (09:00) in the ratio of 3 : 1, respectively, for copulation. Vaginal smear test was performed for each female rat every 24 h and the pregnancy were confirmed by the existence of diestrum stage for three consecutive days. The day of confirmation of pregnancy was designated as GND 3 and the pregnant rat was isolated into a separate cage. The dose of LTG was arrived from the maximum recommended human dose (500 mg) in the management of epilepsy (http://www.drugs.com/).

Pregnant rats were randomized into 4 groups (6 in each) based on stratified body weight. Group-I received vehicle (0.5% CMC; 10 mL/kg, p.o.); Group-II, III, and IV received LTG at 11.5, 23, and 46 mg/kg, p.o., respectively (three-dose regimen was selected to investigate the dose dependent response). Weekly body weight and cumulative feed and water consumption were measured in dam. LTG was administered from GND 3 and continued till 11 days following parturition. Pregnant rats were allowed to deliver and wean their pups until PND 21. Male and female offspring were separated from dam on day 22 following delivery.

### 2.4. Physical Parameters

Length of gestation in dam and viability index of the pups were recorded. Litter sizes in each group were restricted to eight pups (4/sex). Body weight gain was measured on postnatal days once in a week till the study termination. The day of occurrence for pinna detachment, incisor eruption, eye opening, vaginal opening, and testes descent was also recorded.

### 2.5. Behavioural Function in Male and Female Offspring

Offspring were allowed to mature for 90 days with free access to food and water* ad libitum*. On PND 91, the animals were subjected to behavioural assessment.

#### 2.5.1. Anxiety-Elevated Plus Maze

Anxiety was assessed in offspring using elevated plus maze [15]. Elevated plus maze was constructed of black painted wood with two open arms (50 × 10 × 1 cm) and two closed arms (50 × 10 × 40 cm) extending from a common central platform (10 × 10 cm) and elevated to 45 cm above floor level. Experiments were carried out in a sound-attenuated, temperature-controlled room and illuminated by two 60 w fluorescent light. On PND 91, rats were individually placed in the center of the maze facing open arm. Number of entries into open and closed and time spent in open and closed arms were recorded for a period of 5 min [16–19].

#### 2.5.2. Locomotor Function-Open Field Exploratory Test

Locomotor behavioural assessment in experimental groups was performed using open field exploratory test [20]. Open field apparatus consisted of a plywood floor (96 × 96 cm) with high walls. The entire apparatus was painted black except for 6 mm thick white lines which divided the floor into 16 squares. On PND 91, each animal was placed at one corner of the apparatus and observed for next 5 min. The number of squares crossed, immobility period (in seconds), and number of rearing and grooming were recorded.

#### 2.5.3. Learning- and Memory-Radial Arm Maze

Radial arm maze was made of black painted wood finished with a polyurethane coating and consisted of 8 arms with a center platform of 60 cm in diameter. The arms were 80 cm long and 15 cm wide. A disposable plastic cup was placed at each end of the arm to reinforce the sweet smash and all arms were baited with either a food cup or an empty cup. The entire radial arm maze was elevated 50 cm off the floor. Visual cues were located throughout the room to provide spatial cues. The maze was wiped down with 70% alcohol between each trail. The offspring were food-restricted to 85–90% and maintained for the duration of training and testing period.

The maze task was divided into three phases: adaptation, training, and retention phases. In adaptation phase, the rats were allowed to explore the radial arm maze by 3 × 5 min trials. During this period, all of the arms of the maze were baited with the food so that the rats received experience in gaining a feed reinforcement by completely traversing an arm and reaching into the food cup. Time taken by the animal to move from the starting point (lag period), number of working memory errors, and time taken by the animal to munch the food in all arms were recorded. During the training period, four alternative arms were baited with food. The same arms were remained baited for all training and retention trials. Three 5-minute trials were conducted to each animal for ten days. The trial was terminated when the rat has entered and eaten from all the four baited arms. At the end of the three trials of each day, the rat was returned to its home cage. In retention period, the animal was tested 24 h after the final training trials. They received three trials as described under the training procedure. Lag time, number of reference memory errors, working memory errors, correct entries, and total time taken by the animal to munch the food were recorded [21].

### 2.6. GABA-A and GABA-B mRNA Expression by Reverse Transcriptase-Polymerase Chain Reaction (RT-PCR)

After behavioural assessment, all the experimental animals were euthanized using CO_2_ exposure and cortical brain structures were collected for GABA-A and GABA-B mRNA expression. Briefly, the total RNA was extracted using TRIzol Reagent (Sigma, USA). After homogenization, the tubes were incubated for 10 min and centrifuged at 1000 rpm for 5 min. 200 *μ*L of chloroform was added to the supernatant, allowed to incubate for 5 min at room temperature, and centrifuged at 12000 rcf for 20 min. Then 500 *μ*L of isopropyl alcohol was added to the supernatant to precipitate total RNA and centrifuged at 12000 rcf for 15 min following the incubation period of 10 min. Supernatant was decanted carefully and pellet was washed thrice with 75% alcohol and centrifuged at 12000 rcf for 15 min and the pellet was dried. The pellet was resuspended in RNase-free water and stored in −80°C until use. The isolated RNA was allowed to undergo reverse transcription and polymerization reaction to get cDNA using RT-PCR master cycler gradient (Genet Bio, Korea). The gene expression was analyzed using the bands formed in agarose gel electrophoresis, captured using Gel documentation unit (Vilber Laumar, Germany) and quantified by Bio ID software.

Primers sequence used were as follows: GABA-A: sense, 5′-AAG GAC CCA TGA CAG TGC TC-3′; antisense, 5′-GGC TCC CTT GTC CAC TCA TA-3′. GABA-B: sense, 5′-GCT GGA TGG TTA CCA CAT AG-3′; antisense 5′-GGT CAC AGG AGC AGT GAT G-3′ and *β*-actin: sense, 5′-TGC TGT CCC TGT ATG CCT CT-3′; antisense, 5′-AGG TCT TTA CGG ATG TCA ACG-3′ [22].

### 2.7. Statistical Analysis

Data were expressed as mean ± standard error of mean (SEM). Mean differences between the groups were analysed by one way ANOVA followed by Tukey's multiple comparison as post-hoc test.* P* value ≤0.05 was considered to be significant. Statistical analysis was performed using GraphPad prism 4.0 (San Diego, USA).

## 3. Results

### 3.1. Effect of LTG on Dam Body Weight and Feed and Water Consumption

Administration of LTG, at 46 mg/kg, p.o, produced severe signs of toxicity such as hyperesthesia, vocalisation, recumbency, vaginal bleeding, nasal discharge, and finally death of the dam between GND 15 and 17. A significant decrease in body weight, food, and water consumption was observed in LTG (11.5 and 23 mg/kg, p.o) administered dam on GND 14 (*F*(3,20) = 5.81,  *P* < 0.01; * F*(3,20) = 43.11,  *P* < 0.01 and* F*(3,20) = 15.51,  *P* < 0.01, resp.) and 21 (*F*(2,15) = 6.74,  *P* < 0.01; * F*(2,15) = 55.23,  *P* < 0.01 and* F*(2,15) = 13.08,  *P* < 0.01, resp.) when compared to control (Table 1).

### 3.2. Effect of LTG on Physical Parameters in Offspring

**Figure 1:**
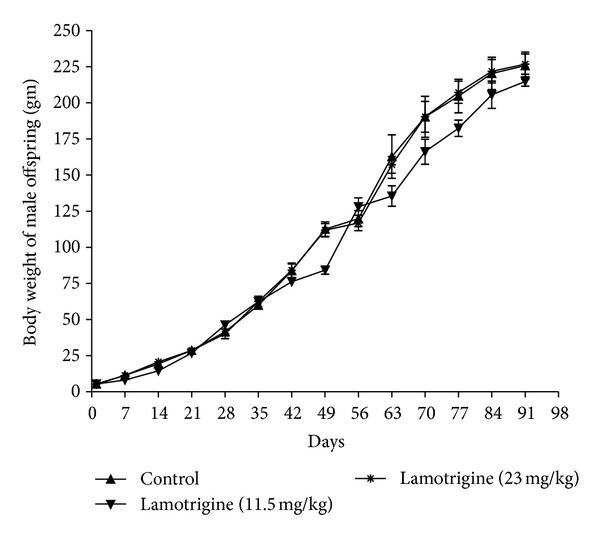
Effect of LTG on body weight of male offspring.

**Figure 2:**
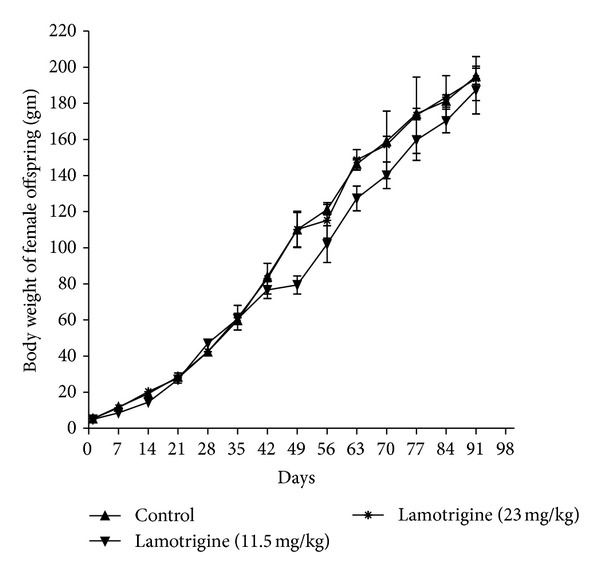
Effect of LTG on body weight of female offspring.

LTG (11.5 and 23 mg/kg, p.o) significantly increased (*F*(2,15) = 23.83,  *P* < 0.01) the gestational period when compared to control rats. No significant difference in litter size between the groups was observed. However, a nonsignificant decrease in pups viability index was observed in LTG administered group when compared to control group (Table 2). LTG (11.5 and 23 mg/kg, p.o) produced a significant delay in incisor eruption in both male (*F*(2,15) = 6.96,  *P* < 0.05) and female (*F*(2,9) = 6.36,  *P* < 0.05) offspring when compared to control offspring. A similar observation was recorded in vaginal opening in female offspring (*F*(2,9) = 286.5,  *P* < 0.01). There were no significant differences in the day of pinna detachment, eye opening, and testes decent observed between LTG and control offspring (Table 3). No significant difference in body weight was observed in both male and female offspring in comparison to control offspring throughout the study (Figures 1 and 2).

### 3.3. Effect of LTG on Offspring Behaviour

#### 3.3.1. Anxiety-Elevated Plus Maze

LTG male showed no significant difference in number of entries and time spent between open and closed arms. However, LTG female (23 mg/kg, p.o) showed a significant increase in number of entries (*F*(2,9) = 5.14,  *P* < 0.05) and time spent (*F*(2,9) = 58.57,  *P* < 0.01) in open arms and decreased number of entries (*F*(2,9) = 20.88,  *P* < 0.01) and time spent (*F*(2,9) = 58.57,  *P* < 0.01) in closed arms when compared to control rats (Table 4).

#### 3.3.2. Locomotor Function-Open Field Exploratory Test

No significant difference was observed in number of squares crossed, immobility period, and between the experimental groups. However, a significant (*F*(2,9) = 4.07,  *P* < 0.05) increase in rearing behaviour was observed in LTG (23 mg/kg, p.o) female offspring when compared to control group (Table 5).

#### 3.3.3. Learning- and Memory-Radial Arm Maze

There was no significant difference in lag period, number of reference and working memory errors, number of correct entries, and total time taken by the animal to munch the food recorded between the experimental groups (Table 6).

### 3.4. Effect of LTG on GABA-A and GABA-B mRNA Expressions

**Figure 3:**
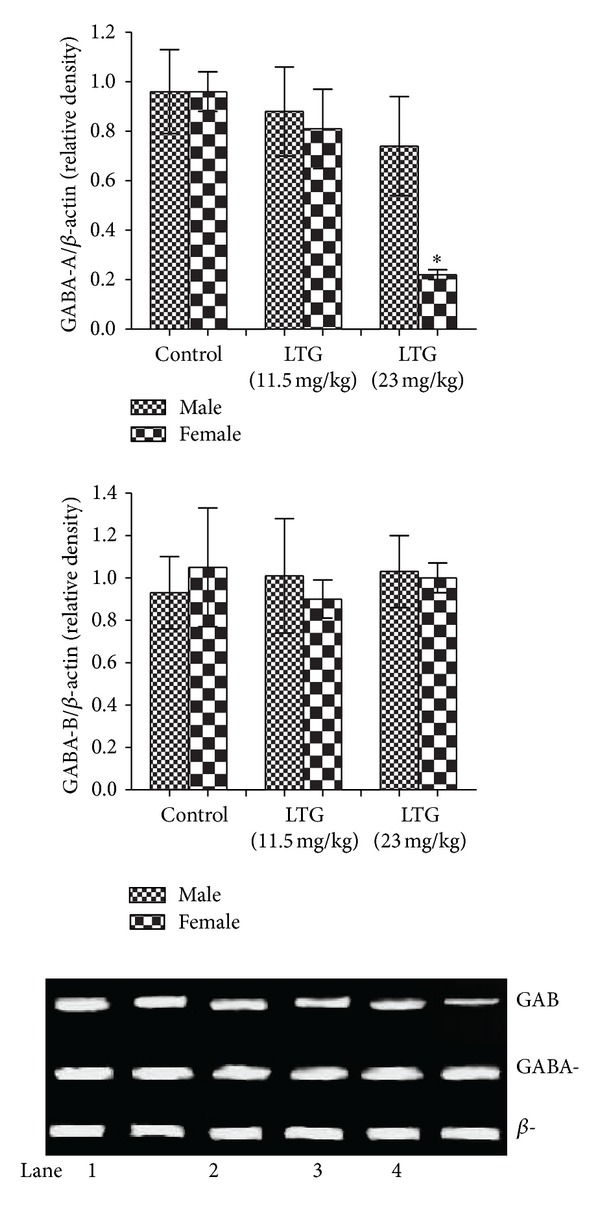
Effect of LTG on GABA receptors in male and female offspring. Lane 1- and 2-Control (male and female offspring, resp.); Lane 3- and 4-LTG: 11.5 mg/kg, p.o (male and female offspring, resp.); Lane 5- and 6-LTG: 23 mg/kg, p.o (male and female offspring, resp.). *denotes *P* ≤ 0.05 versus control group.

mRNA expression of GABA-A was found to be significantly (*F*(2,3) = 17.07, *P* < 0.05) downregulated in LTG female offspring (23 mg/kg, p.o) when compared to counterparts; whereas, no difference in GABA-B expression was observed between the experimental groups (Figure 3).

## 4. Discussion

Use of antiepileptic drugs, such as LTG and levetiracetam, during pregnancy has become challenging these days as they have toxic effects on the developing embryo. Several investigations in rat and rabbit models revealed that LTG crosses the placenta [23]. Administration of LTG in the form of single therapy or polytherapy is at a high risk of developing signs and symptoms of fetal toxicity [24]. The present study demonstrates that prenatal exposure of LTG in rats produced severe signs of toxicity in dam and gender differential behavior in female offspring.

Various studies demonstrated that prenatal LTG exposure at a dose of 250 mg/kg (half the human equivalent dose) increases stillbirth and postnatal death of the offspring [25, 26]. In the current study, gestational LTG exposure at 46 mg/kg produced severe toxic signs such as hyperesthesia, vocalisation, recumbency, vaginal bleeding, nasal discharge, and finally death of the dam. Sidhu et al. [27] showed that lamotrigine administration increases the follicle stimulating hormone (FSH) and luteinizing hormone (LH) which in turn stimulated estrogen secretion. Increased estrogen secretion results in maturation of graaffian follicle followed by ovulation which leads to embryo detachment. This may be the possible reason for the observed fetal death in the present study.

In addition, LTG induces the secretion of parathyroid hormone (PTH) thereby modulating calcium homeostasis leading to osteoporosis [28]. It is well known that osteogenesis of the embryo occurs at GND 15–21 in rats. Increased serum calcium levels by PTH reduce the fetal osteogenesis during embryonic development which could also be one of the possible factors for observed fetal death at GND 15–17 in LTG high dose administered rats. Decreased body weight gain and feed and water intake of dam treated with LTG revealing its possible maternal toxic effects. Further, delayed vaginal opening and incisors eruption in offspring of LTG lower doses group might also be due to its maternal toxic effects [13].

On the other hand, it is reported that LTG administration along with the estrogen-containing hormonal contraceptives reduces the serum LTG concentration. Hence the dose has to be increased when administered with such contraceptives to maintain its level of action [29]. In addition, increased estrogen level induces FSH and secretes progesterone and testosterone by feedback control process thereby lengthening the gestational period. Although progesterone and testosterone levels were not measured in dams, the increased gestational length observed in our study of rats who administered LTG at lower doses may be due to impaired luteolysis and increased progesterone levels.

Earlier studies have shown that blockade of GABA-A receptor in rat brain induces hyperexcitability/anxiolytic behaviour in elevated plus maze [30, 31]. Progesterone and estrogen induce anxiolytic behaviour in C57BL/6 female mice [32]. Further, estrogen suppresses GABA-A receptor expression thereby slowing down the GABA mediated inhibition [33]. In the present study, the observed decrease in GABA-A expression and hyperactivation behaviour (as evidenced by the elevated plus maze and open field test) may be due to the overactivation of G-protein-coupled receptor 30 (GPR30) by estrogen hormone in the female offspring. Put together, the observations showed that the anxiolytic behaviour in female offspring but not the male may be due to the defect in GABA-A expression and also the alterations in GPR30 mediated estrogen secretion.

## 5. Conclusion

In conclusion, the present study demonstrates the potential untoward effects of LTG in pregnant rats and also its influence on the postnatal development and gender based differential behavioural effects in offspring. Our lab is further investigating the mechanisms and reason for these untoward effects so as to utilise the therapeutic benefits of LTG in a safer way.

## Figures and Tables

**Table 1:** Effect of LTG on dam body weight and feed and water consumption.

Treatment	Control	LTG (mg/kg, p.o)		
		11.5	23	46
Dam body weight (g)				
GND 3	182.00 ± 6.00	165.00 ± 1.20	169.83 ± 3.56	183.69 ± 7.82
GND 7	187.00 ± 8.50	171.67 ± 1.64	168.83 ± 4.12	180.50 ± 4.85
GND 14	227.50 ± 13.25	188.33 ± 5.60*	185.53 ± 5.64*	178.33 ± 9.91**
GND 21	242.00 ± 8.50	201.66 ± 12.88*	191.00 ± 9.16**	—
Cumulative feed consumption (g/dam)				
GND 3–6	40.75 ± 2.37	37.00 ± 3.14	39.29 ± 1.37	41.37 ± 3.48
GND 7–14	86.00 ± 1.14	60.28 ± 4.26**	58.00 ± 2.09**	47.56 ± 0.96**
GND 15–21	88.50 ± 1.86	65.25 ± 2.50**	60.80 ± 1.52**	—
Cumulative water consumption (mL)				
GND 3–6	43.39 ± 0.31	41.25 ± 2.32	39.77 ± 0.29	44.56 ± 1.55
GND 7–14	102.50 ± 0.59	96.54 ± 1.56*	95.80 ± 1.09**	91.00 ± 1.33**
GND 15–21	95.50 ± 1.08	86.59 ± 2.03**	85.80 ± 1.17**	—

*n* = 6/group. Values are expressed in mean ± SEM. Significance with Tukey's test following one way ANOVA is indicated as *P ≤ 0.05 and **P ≤ 0.01 versus control group; GND: gestational day; LTG: lamotrigine.

**Table 2:** Effect of LTG on length of gestation, total number of litters, and its viability index.

Group	Treatment	Gestational length (Days)	Total number of litters	Viability index (%)
I	Control	19.33 ± 0.33	10.00 ± 0.58	100.00 ± 0.00
II	LTG (11.5 mg/kg, p.o)	22.25 ± 0.47**	6.75 ± 1.49	80.20 ± 15.90
III	LTG (23 mg/kg, p.o)	22.00 ± 0.00**	8.33 ± 0.67	81.48 ± 13.36
IV	LTG (46 mg/kg, p.o)	—	—	—

*n* = 6/group. Values are expressed in mean ± SEM. Significance with Tukey's test following one way ANOVA is indicated as *P ≤ 0.05 and **P ≤ 0.01 versus control group.

**Table 3:** Effect of LTG on physical growth of male and female offspring.

Group	Treatment	Day of pinna detachment		Day of incisor eruption		Day of eye opening		Day of testes descent	Day of vaginal opening
		Male	Female	Male	Female	Male	Female		
I	Control	4.33 ± 0.33	4.67 ± 0.33	7.33 ± 0.33	7.33 ± 0.33	16.67 ± 0.33	16.00 ± 0.33	6.33 ± 0.33	32.67 ± 0.33
II	LTG (11.5 mg/kg, p.o)	5.33 ± 0.33	5.33 ± 0.33	9.33 ± 0.33*	9.00 ± 0.57	15.66 ± 0.66	15.00 ± 0.67	6.23 ± 1.67	40.57 ± 0.33**
III	LTG (23 mg/kg, p.o)	5.30 ± 0.29	5.66 ± 0.33	9.50 ± 0.64*	9.33 ± 0.33*	15.33 ± 0.33	14.33 ± 0.33	9.00 ± 0.00	54.00 ± 1.00**

*n* = 8/group (4/sex). Values are expressed in mean ± SEM. Significance with Tukey's test following one way ANOVA is indicated as *P ≤ 0.05 and **P ≤ 0.01 versus control group.

**Table 4:** Effect of LTG on anxiety behaviour in male and female offspring.

Group	Treatment	Sex	Number of entries		Time spent (sec)	
			Open	Closed	Open	Closed
I	Control	Male	2.50 ± 0.50	10.25 ± 1.18	112.25 ± 12.94	187.75 ± 12.94
		Female	3.00 ± 1.34	11.00 ± 0.84	122.40 ± 9.92	177.60 ± 9.92
II	LTG (11.5 mg/kg, p.o)	Male	2.08 ± 0.94	7.03 ± 1.25	133.72 ± 28.13	166.28 ± 28.13
		Female	4.67 ± 1.00	9.67 ± 1.16	97.92 ± 16.89	202.08 ± 16.89
III	LTG (23 mg/kg, p.o)	Male	2.83 ± 0.48	8.00 ± 0.73	147.33 ± 10.97	152.67 ± 10.97
		Female	10.50 ± 2.50*	3.50 ± 0.50**	262.50 ± 4.50**	37.50 ± 4.50**

*n* = 8/group (4/sex). Values are expressed in mean ± SEM. Significance with Tukey's test following one way ANOVA is indicated as *P ≤ 0.05 and **P ≤ 0.01 versus control group.

**Table 5:** Effect of LTG on locomotor function in male and female offspring.

Group	Treatment	Sex	Number of squares crossed	Immobility period (sec)	Number of rearing	Number of grooming
I	Control	Male	89.25 ± 7.45	100.25 ± 21.99	27.25 ± 2.43	8.25 ± 1.25
		Female	109.60 ± 7.78	72.00 ± 11.36	37.60 ± 4.95	7.40 ± 0.75
II	LTG (11.5 mg/kg, p.o)	Male	87.00 ± 5.29	74.50 ± 13.82	28.00 ± 1.34	6.17 ± 0.54
		Female	109.50 ± 6.50	81.42 ± 17.32	46.50 ± 3.50	9.00 ± 3.00
III	LTG (23 mg/kg, p.o)	Male	77.52 ± 16.40	112.03 ± 41.11	20.85 ± 3.85	6.12 ± 1.07
		Female	113.58 ± 4.64	50.00 ± 10.00	51.75 ± 1.00*	5.08 ± 0.63

*n* = 8/group (4/sex). Values are expressed in mean ± SEM. Significance with Tukey's test following one way ANOVA is indicated as *P ≤ 0.05 versus control group.

**Table 6:** Effect of LTG on learning and memory function in male and female offspring.

Group	Treatment	Sex	Lag period	Number of reference memory errors	Number of working memory errors	Number of correct entries	Total time taken to munch the food
I	Control	Male	1.00 ± 0.00	1.83 ± 0.80	0.42 ± 0.12	2.25 ± 0.74	82.50 ± 19.04
		Female	0.33 ± 0.20	0.73 ± 0.37	0.20 ± 0.13	2.53 ± 0.51	88.93 ± 15.81
II	LTG (11.5 mg/kg, p.o)	Male	1.06 ± 0.41	2.67 ± 0.44	0.28 ± 0.08	1.22 ± 0.43	137.11 ± 15.54
		Female	0.32 ± 0.10	2.67 ± 0.74	1.17 ± 0.50	1.33 ± 0.89	113.17 ± 15.00
III	LTG (23 mg/kg, p.o)	Male	1.25 ± 0.33	3.00 ± 0.74	0.71 ± 0.17	1.25 ± 0.53	141.25 ± 15.68
		Female	0.47 ± 0.22	1.46 ± 0.72	0.75 ± 0.43	2.33 ± 0.69	63.71 ± 19.24

*n* = 8/group (4/sex). Values are expressed in mean ± SEM.

## Abbreviations

AED: Antiepileptic drug
LTG: Lamotrigine
GND: Gestational day
PND: Postnatal day
GABA-A: *γ*-Aminobutyric acid A receptor
GABA-B: *γ*-Aminobutyric acid B receptor
CMC: Carboxymethyl cellulose
IAEC: Institutional Animal Ethics Committee
RT-PCR: Reverse transcriptase polymerase chain reaction
SEM: Standard error of mean
PTH: Parathyroid hormone
FSH: Follicle stimulating hormone
LH: Luteinizing hormone
GPR30: G-protein-coupled receptor 30.
